# Dominant Incidence of Multidrug and Extensively Drug-Resistant Specific *Mycobacterium tuberculosis* Clones in Osaka Prefecture, Japan

**DOI:** 10.1371/journal.pone.0042505

**Published:** 2012-08-31

**Authors:** Aki Tamaru, Chie Nakajima, Takayuki Wada, Yajun Wang, Manabu Inoue, Ryuji Kawahara, Ryoji Maekura, Yuriko Ozeki, Hisashi Ogura, Kazuo Kobayashi, Yasuhiko Suzuki, Sohkichi Matsumoto

**Affiliations:** 1 Department of Infectious Diseases, Osaka Prefectural Institute of Public Health, Osaka, Osaka, Japan; 2 Department of Bacteriology, Osaka City University Graduate School of Medicine, Osaka, Japan; 3 Division of Global Epidemiology, Hokkaido University Research Center for Zoonosis Control, Sapporo, Hokkaido, Japan; 4 Department of Microbiology, Osaka City Institute of Public Health and Environmental Sciences, Osaka, Japan; 5 Department of Internal Medicine, National Hospital Organization National Toneyama Hospital, Toyonaka, Osaka, Japan; 6 Department of Food and Nutrition, Sonoda Women’s University, Amagasaki, Hyogo, Japan; 7 Department of Virology, Osaka City University Graduate School of Medicine, Osaka, Japan; 8 Departement of Immunology, National Institute of Infectious Diseases, Shinjyuku-ku, Tokyo, Japan; 9 Japan Science and Technology Agency/Japan International Cooperation Agency-Science and Technology Research Partnership for Sustainable Development, Chiyoda-ku, Tokyo, Japan; Hopital Raymond Poincare - Universite Versailles St. Quentin, France

## Abstract

Infection and transmission of multidrug-resistant *Mycobacterium tuberculosis* (MDR-Mtb) and extensively drug-resistant *M. tuberculosis* (XDR-Mtb) is a serious health problem. We analyzed a total of 1,110 Mtb isolates in Osaka Prefecture and neighboring areas from April 2000 to March 2009. A total of 89 MDR-Mtb were identified, 36 (48.5%) of which were determined to be XDR-Mtb. Among the 89 MDR-Mtb isolates, 24 (27.0%) phylogenetically distributed into six clusters based on mycobacterial interspersed repetitive units-various number of tandem repeats (MIRU-VNTR) typing. Among these six clusters, the MIRU-VNTR patterns of four (OM-V02, OM-V03, OM-V04, and OM-V06) were only found for MDR-Mtb. Further analysis revealed that all isolates belonging to OM-V02 and OM-V03, and two isolates from OM-V04 were clonal. Importantly such genotypes were not observed for drug-sensitive isolates. These suggest that few but transmissible clones can transmit after acquiring multidrug resistance and colonize even in a country with a developed, well-organized healthcare system.

## Introduction

The control of tuberculosis (TB) has become increasingly more urgent because of the emergence of multidrug-resistant tuberculosis (MDR-TB) and extensively drug-resistant tuberculosis (XDR-TB). These two forms of TB are difficult to manage and treat, and known for producing high morbidity and mortality [1], [2]. Multidrug-resistant *Mycobacterium tuberculosis* (MDR-Mtb) is resistant to at least both isoniazid (INH) and rifampin (RFP), while extensively drug-resistant *M. tuberculosis* (XDR-Mtb) is resistant to both INH and RFP, in addition to any of the second-line injectable drugs (capreomycin, kanamycin [KM] or amikacin) and fluoroquinolone [3], [4]. In principle, these resistant *M. tuberculosis* (Mtb) strains are thought to arise through the selection of mutated strains in response to inadequate chemotherapy [1], [2], [5], [6]. It is generally believed that transmission frequency of MDR/XDR-Mtb is lower comparing to drug-susceptible strains, however, exogenous infection with MDR/XDR-Mtb was reported, especially in countries with high human immunodeficiency virus (HIV) infection rates [6]–[8].

Several studies that genotyped MDR-Mtb isolates have shown that some are genetically related, forming phylogenetic clusters with identical genotypes [9]–[12]. Most of these reports showed that MDR-Mtb genetic clusters consist of isolates that belong to the Beijing genotype [7], [10], [12], suggesting a high tendency to become MDR-Mtb Beijing strains. Beijing strains are endemically predominant in East Asia [13] and are epidemically spreading in isolated regions such as Cuba, Eastern Europe, sub-Saharan Africa, and Vietnam [14]. Overall, Beijing strains of Mtb represent approximately 10% of MDR-Mtb isolates worldwide [14].

In 2009, Japan had intermediate TB incidence (19.0/100,000) [15] and a low HIV infection rate (new HIV infections, 0.8/100,000) (calculated from data in [16]). The prevalence of new MDR-TB cases in Japan was 0.7% and new XDR-TB cases was 0.2% [17]. Using this statistical ratio, it is estimated that approximately 170 new MDR-TB cases including approximately 50 XDR-TB cases occur every year. Moreover, greater than 70% of all Mtb isolated in Japan were of the Beijing genotype [18].

Osaka Prefecture has historically had the highest endemic rate of TB in Japan since 1991. The rate of TB incidence in Osaka in 2009 was determined to be 31.5/100,000 (16), while maintaining a low HIV infection rate (1.9/100,000) (calculated from data in [16]). Osaka Prefecture is located in central Japan and has an area of 1,989 km^2^. It is a highly industrialized area including Osaka, the third most populous city in Japan, and over 8 million people have lived in Osaka Prefecture since 1973. In Osaka, both MDR-TB and XDR-TB cases have been reported, but little is known about the transmission of MDR/XDR-Mtb and the prevalence of Beijing lineage genotypes among MDR-Mtb isolates.

In the present study, we characterized a total of 1,110 Mtb isolates obtained from TB patients in Osaka prefecture and a neighboring area utilizing mycobacterial interspersed repetitive units-various number of tandem repeats (MIRU-VNTR) typing to investigate transmission of MDR-TB. Our data revealed that MDR/XDR-Mtb clones displayed genotypes that are not typically seen in drug-susceptible isolates and these strains are colonizing and spreading in Osaka Prefecture and its neighboring area.

## Materials and Methods

### Mtb Isolates

We analyzed 89 MDR-Mtb and 1021 non MDR-Mtb isolates collected in Osaka Prefectural Institute of public health. Eighty-nine MDR-Mtb isolates, which were delivered from three hospitals having tuberculosis ward in Osaka prefecture, were isolated from MDR-TB patients in Osaka Prefecture (except Osaka city and southeast area of Osaka prefecture) and neighboring local governments from April 2000 to March 2009. Of 1021 non MDR-Mtb isolates of which genetic diversity and/or genotypes were compared with those of MDR-Mtb, 471 isolates were sent to the institute for routine examinations (identifications of *Mtb*, drug susceptibility, and/or determination of the source of TB outbreaks) by Osaka prefectural health care centers from April 2000 to March 2007 and by neighboring local governments from April 2000 to March 2009. The remaining 550 were isolates from newly diagnosed TB patients in Osaka prefecture (except Osaka city and southeast area of Osaka prefecture) and sent to the institute for population-based molecular epidemiology of TB (population-based non MDR-Mtb). All Mtb isolates were confirmed whether they were MDR-Mtb or not in the laboratories of the hospitals, commercial laboratories, or Osaka Prefectural institute of public health. Although we could not fully obtain patient data including age, sex, residence area, year of case reported, and history of TB, duplication of patients of the isolates were avoided by the unified patients’ identification numbers and the names of the patients.

### Drug Susceptibility Test

To determine the ratio of XDR-Mtb for MDR-Mtb, we performed drug susceptibility tests of MDR-Mtb. When MDR-Mtb isolates were grown on Ogawa medium, 15 of the 89 isolates provided failed to grow on this medium because they perished. Therefore, for the remaining 74, minimum inhibitory concentrations (MICs) for INH, RFP, KM, streptomycin (SM), ethambutol (EB), and the fluoroquinolones, levofloxacin (LVFX), spafloxacin (SPFX), and ciprofloxacin (CPFX) were determined using the micro-dilution test BrothMIC MTB I (Kyokuto, Tokyo). Mtb isolates resistant to INH, RFP, KM and at least one fluoroquinolone were defined as XDR-Mtb.

### DNA Extraction and Genotyping

Chromosomal DNA was extracted by combining chloroform extraction and mechanical disruption [19] from cultures grown on Ogawa medium or MGIT liquid medium for all 89 isolates. DNA was extracted from all 1110 isolates and genotyped using the MIRU-VNTR type testing [18], [20] for 26 loci to identify transmission routes. Single-locus variants involving QUB4120, QUB11a, QUB11b, and QUB26 were regarded as identical in this study. All 89 MDR-Mtb isolates and 550 population-based non MDR-Mtb were examined by PCR to determine the frequency of the Beijing lineage [21]. Isolates determined as genetically related to the Beijing lineage were further classified into ancient and modern sub-lineages by PCR [22], [23].

### Sequence Analysis

MDR-Mtb isolates showing identical genetic patterns were analyzed by their SNPs for *katG*, *inhA*, *rpoB*, *rrs*, and *gyrA*. PCR products were amplified using the following primers: *katG*-Fw, CGCAGCGAGAGGTCAGTGGCCAG; *katG*-Rv, ATGGCCATGAACGACGTCGAAAC; *inhA-*Fw, TCACACCGACAAACGTCACGAGC; *inhA*-Rv, AGCCAGCCGCTGTGCGATCGCCA; *rpoB*-Fw, CAGGACGTGGAGGCGATCAC; *rpoB*-Rv, GAGCCGATCAGACCGATGTTGG; *rrs*-Fw, TCACCATCGACGAAGCTCCG; *rrs*-Rv, CTAGACGCGTCCTGTGCATG; *gyrA*-Fw, AGCGCAGCTACATCGACTATGCG; and *gyrA*-Rv, CTTCGGTGTACCTCATCGCCGCC. All amplified products were purified and sequenced using the Big Dye Terminator Cycle Sequencing Kit (Applied Biosystems Inc., Japan) on a ABI Prism 3100 Genetic Analyzer (Applied Biosystems Inc.).

## Results

### Identification of MDR/XDR-Mtb and Drug Sensitivity Patterns

The drug susceptibility patterns of 74 MDR-Mtb isolates were identified to investigate the prevalence of XDR-Mtb among MDR-Mtb isolates (Table 1) and 36 (48.6%) were determined to be XDR-Mtb. Of these 36 XDR-Mtb, 25 (33.8%) were determined to be resistant to all eight drugs tested in this study. Of the 38 MDR-Mtb isolates, eight were resistant only to INH and RFP (10.8%), while the remaining 30 were resistant to INH, RFP, and either one or more second-line injectable drugs or one or more fluoroquinolone.

**Table 1:** Drug resistance patterns of 74 MDR-TB isolates.

MDR or XDR	Total number of isolates (%)*	Drug resistance pattern								Number of isolates (%)*
XDR	36 (48.6)	INH	RFP	KM	SM	EB	LVFX	CPFX	SPFX	25 (33.8)
		INH	RFP	KM		EB	LVFX	CPFX	SPFX	8 (10.8)
		INH	RFP	KM			LVFX	CPFX	SPFX	1 (1.4)
		INH	RFP	KM			LVFX	CPFX		1 (1.4)
		INH	RFP	KM	SM		LVFX	CPFX	SPFX	1 (1.4)
MDR	38 (51.4)	INH	RFP		SM	EB	LVFX	CPFX	SPFX	4 (5.4)
		INH	RFP			EB	LVFX	CPFX	SPFX	4 (5.4)
		INH	RFP		SM		LVFX	CPFX	SPFX	1 (1.4)
		INH	RFP		SM			CPFX	SPFX	1 (1.4)
		INH	RFP		SM	EB		CPFX		1 (1.4)
		INH	RFP	KM	SM	EB				6 (8.2)
		INH	RFP		SM	EB				3 (4.1)
		INH	RFP			EB				5 (6.8)
		INH	RFP		SM					4 (5.4)
		INH	RFP	KM						1 (1.4)
		INH	RFP							8 (10.8)

* Percentage of MDR-TB isolates determined drug sensitivity.

### Comparison of the Genetic Diversity of MDR-Mtb and Non MDR-Mtb

To bring out the genetic aspects of MDR-Mtb, we compared the cluster formation resulting from MIRU-VNTR genotyping and the frequency of Beijing lineage with those of population-based non MDR-Mtb (Table2). Among 89 MDR-Mtb isolates, 24 (27.0%) isolates were classified into six identical clusters. In population-based non MDR-Mtb, sixty-five identical clusters were formed from 216 (39.3%) isolates. The ratio of cluster-forming isolates in MDR-Mtb was significantly lower than it in population-based non MDR-Mtb.

**Table 2:** Comparison of the genetical diversity of MDR-Mtb and non MDR-Mtb.

	The number of clusters	The number of isolates		
		included in any clusters	belonging to Beijing Lineage	belonging to ancient sublineage
MDR-Mtb	6	24	79	60
		27.0%	88.8%	75.9%**
non MDR-Mtb	65	216*	425*	328
		39.3%	77.3%	77.2%**

* significant difference (P<0.05, Fisher’s exact test).

** percentage for Beijing isolates.

The frequency of the Beijing Lineage was 88.8% in MDR-Mtb and 77.3% in population-based non MDR-Mtb, showing significantly higher frequency of the Beijing Lineage in MDR-Mtb isolates. The Beijing lineage can be divided into ancient and modern sub-lineage by the insertion of IS*6110* into the NTF region of the genome [22]. About the ratio of ancient sub-lineage and modern, the significant difference was not seen in MDR-Mtb isolates and population-based non MDR-Mtb (75.9% and 77.2%, respectively).

### Comparison of MIRU-VNTR Patterns between MDR- andnon-MDR-Mtb Isolates

The MIRU-VNTR patterns of the 6 identical clusters constructed from MDR-Mtb isolates OM-V02, OM-V03, OM-V04, OM-V06, OM-V12, and OM-V32 were shown in Table 3. To verify these MIRU-VNTR pattern were specific to MDR-Mtb isolates or not, we genotyped not only population-based non MDR-Mtb isolates but also other 471 non MDR-Mtb isolates (total 1021 non MDR-Mtb isolates were genotyped) and all MDR-Mtb clusters and non-MDR-Mtb clusters with incidence rates of more than 1.0% per total isolates are presented in Table 3. Importantly four clusters, OM-V02, OM-V03, OM-V04, and OM-V06, consisted of only MDR-Mtb isolates and none of the 1,021 non-MDR-Mtb isolates. These four clusters together were designated as a MDR-Mtb-specific cluster (MDR-Mtb SC). In particular, the incidence rate of OM-V02 (1.1% of 1,110 isolates) was the highest among the MDR-Mtb SC and equal to that of OM-V801, the sixth largest non-MDR-Mtb cluster.

**Table 3:** MIRU-VNTR profiles of phylogenetic clusters.

Cluster type*	Cluster No.	Number of alleles in the MIRU-VNTR loci†																										number of isolates in cluster			Beijing sublineage
																												MDR	non MDR	(%)‡	
MDR-TB	OM-V02	2	1	3	8	7	3	5	2	4	4	4	3	4	3	4	3	5	2	4	4	7	4	9	7	8	10	11	0	(1.1)	ancient
	OM-V03	2	3	3	5	7	3	5	3	3	4	4	3a	2	3	4	2	4	3	3	2	10	4	25	6	8	4	4	0	(0.4)	ancient
	OM-V04	3	3	3	5	7	3	5	3	3	2	4	3	4	3	2	3	4	3	3	4	5	4	8	1	7	9	3	0	(0.3)	ancient
	OM-V06	2	3	3	5	7	3	5	3	3	3	4	3	4	4	3	3	3	3	3	4	7	4	5	5	8	10	2	0	(0.2)	modern
Mix C	OM-V12	2	3	4	5	7	2	5	3	3	4	4	3	3	3	4	3	5	4	3	5	7	2	8	8	2	11	2	7	(0.8)	ancient
	OM-V32	2	3	3	5	7	3	5	3	3	4	4	3	4	4	4	3	3	3	3	4	7	4	8	8	8	9	2	11	(1.2)	modern
	OM-V29	2	3	3	5	7	3	5	3	3	4	4	3	4	4	4	3	3	3	3	4	7	4	5	5	8	8	1	30	(2.8)	modern
	OM-V05	2	3	3	5	7	3	5	3	3	4	4	3a	3	3	4	3	4	3	3	2	10	4	5	5	6	8	1	16	(1.5)	ancient
	OM-V031	2	3	4	5	7	3	5	3	3	4	4	3	3	3	4	3	5	4	3	5	7	2	8	7	2	9	1	6	(0.6)	ancient
	OM-V091	2	3	3	5	7	3	5	3	3	4	4	3	4	3	4	3	5	2	3	4	7	4	5	7	10	8	1	4	(0.5)	ancient
	OM-V021	2	3	3	5	4	3	5	3	3	4	4	3	4	4	4	3	3	3	3	4	7	4	8	6	8	10	1	4	(0.5)	modern
	OM-V070	1	5	1	5	1	3	3	2	2	3	4	3a	2	2	4	3	4	1	3	2	12	2	7	2	9	3	1	3	(0.4)	non Beijing
	OM-V067	2	3	3	5	7	1	5	3	1	4	4	3	4	3	4	3	3	3	3	4	7	4	8	9	8	10	1	3	(0.4)	modern
	OM-V08	2	3	3	5	7	3	4	3	3	4	4	3	4	0	2	3	4	3	3	4	1	6	8	3	8	10	1	2	(0.3)	ancient
	OM-V068	2	3	3	6	5	3	3	1	1	3	3	3	2	1	2	6	3	3	2	4	12	2	2	2	3	2	1	1	(0.2)	non Beijing
	OM-V087	2	1	3	5	7	3	5	3	3	4	4	3	4	3	4	3	5	2	4	4	7	4	7	6	8	11	1	1	(0.2)	ancient
non MDR	OM-V127	2	3	4	5	7	3	5	3	3	4	4	3	3	3	4	3	5	4	3	5	7	2	8	7	2	11	0	13	(1.2)	ancient
	OM-V228	2	3	3	5	7	3	5	3	3	4	4	3a	4	3	4	3	5	3	3	4	7	4	8	7	8	5	0	13	(1.2)	ancient
	OM-V801	2	3	3	5	5	3	3	2	1	2	4	3a	2	1	2	3	3	2	2	4	8	4	2	2	4	2	0	11	(1.1)	ancient

* MDR: clusters consisted of only MDR-TB isolates, Mix C: clusters consisted of MDR-TB isolates and non MDR-TB isolates, non MDR: clusters consisted of only non-MDR-TB isolates for which the ratio of total non MDR-TB isolates is >1%.

† The order of MIRU-VNTR loci is as follows: 0580, 0960, 1644, 2531, 2996, 3007, 3192, 4348, 0802, 2165, 0577, 3239 (3a contains irregular-size alleles), 0424, 1955, 2401, 3690, 4156, 2074, 2372, 3155, 3336, 1895, 2163a, 2163b, 4052, 4120.

‡ Percentage of all TB isolates.

In contrast, two of the six MDR-Mtb clusters, OM-V12 and OM-V32, had the MIRU-VNTR patterns that were also found in non-MDR-Mtb isolates. The other 65 MDR-Mtb isolates formed 10 clusters (OM-V29, OM-V05, OM-V031, OM-V091, OM-V021, OM-V070, OM-V067, OM-V08, OM-V068, and OM-V087) with one or multiple non-MDR-Mtb isolates. The clusters were designated as a mixed cluster (Mix C) type, of which MIRU-VNTR pattern were observed in both MDR and non-MDR Mtb. The remaining 55 MDR-Mtb showed unique MIRU-VNTR patterns.

Different incidence ratios of MDR/non-MDR-Mtb were observed among clusters. For example, OM-V29, which was the largest cluster, consisted of only one MDR-Mtb and 30 non-MDR-Mtb isolates, while two of nine isolates were MDR-Mtb in OM-V12 (the eighth largest cluster) and two of 13 were MDR-Mtb in OM-V32 (the third largest cluster).

### MIC Values and Mutation Patterns of MDR-Mtb SC

To further investigate whether isolates in each cluster in MDR-Mtb SC (OM-V02, OM-V03, OM-V04, and OM-V06) were identical clones, MICs and mutations in the resistance-associated genes were analyzed (Table 4). Although nine of these isolates (Nos. 010, 019, 030, 063, 073, 082, 095, and 004) belonging to OM-V02 were XDR-Mtb and the remaining two (051, 052) were MDR-Mtb, the MICs of these isolates revealed slight variations and all isolates possessed D516V in *rpoB*, S315T in *katG*, and a nucleotide substitution in *rrs* (adenine to guanine at position 1400 in the gene). Nos. 19 and 95 had additional mutations in *katG* and *rpoB*, respectively. Regarding isolates belonging to the OM-V03 cluster, of which MICs and mutations were similar for all isolates, only isolate No. 029 showed a higher MIC value to INH and an additional mutation in *inhA*. Isolate Nos. 002 and 020 in cluster OM-V04 shared the same MIC and mutation patterns. Taken together, these data suggest that OM-V02, OM-V03, and OM-V04 are three clonal strains.

**Table 4:** MIC values for drugs and mutations in *rpoB*, *katG*, *inhA, gryA,* and *rrs* of isolates in the MDR-TB SC.

cluster	MDR isolates No.	MDR or MDR	MIC (ug/ml)								mutations				
			INH	RFP	SM	KM	EB	LVFX	SPFX	CPFX	*rpoB*	*katG*	*inhA*	*rrs*	*gyrA*
OM-V02	005	XDR	8	16	32	>128	64	4	32	4	Asp 516 Val	Ser 315 Thr	none	A 1400 G	Asp 94 Ala
	010	XDR	8	16	64	>128	64	2	1	4	Asp 516 Val	Ser 315 Thr	none	A 1400 G	Asp 94 Ala
	019	XDR	8	32	32	>128	64	2	1	4	Asp 516 Val	Ser 315 Thr, G 944 C	none	A 1400 G	Asp 94 Ala, Thr 95 Thr/Pro
	030	XDR	8	8	16	>128	16	2	1	2	Asp 516 Val	Ser 315 Thr	none	A 1400 G	Asp 94 Ala
	050	MDR	8	>32	32	>128	64	0.5	0.125	0.25	Asp 516 Val	Ser 315 Thr	none	A 1400 G	none
	051	MDR	8	32	64	>128	64	0.5	0.125	0.5	Asp 516 Val	Ser 315 Thr	none	A 1400 G	none
	063	XDR	8	16	32	>128	32	2	1	4	Asp 516 Val	Ser 315 Thr	none	A 1400 G	Asp 94 Ala
	073	XDR	16	8	16	>128	16	4	16	4	Asp 516 Val	Ser 315 Thr	none	A 1400 G	Asp 94 Ala
	082	XDR	8	16	64	>128	64	2	1	2	Asp 516 Val	Ser 315 Thr	none	A 1400 G	Asp 94 Ala
	095	XDR	32	16	>128	8	4	2	1	2	Asp 516 Val, Leu 533 Pro	Ser 315 Thr	none	A 1400 G	Asp 94 Ala
	004	XDR	8	4	16	>128	16	2	1	4	Asp 516 Val	Ser 315 Thr	none	A 1400 G	Asp 94 Ala
OM-V03	013	XDR	8	>32	64	>128	16	8	4	8	Ser 531 Leu	Ser 315 Thr	none	A 1400 G	Asp 94 Gly
	029	XDR	>32	>32	64	>128	16	8	4	8	Ser 531 Leu	Ser 315 Thr	C -15 T	A 1400 G	Asp 94 Gly/Asp
	061*										Ser 531 Leu	Ser 315 Thr	none		Asp 94 Gly
	064	XDR	8	>32	128	>128	16	8	4	16	Ser 531 Leu	Ser 315 Thr	none	A 1400 G	Asp 94 Gly
OM-V04	002	MDR	4	32	4	2	8	2	1	2	Ser 531 Leu	none	C -15 T	none	Asp 94 Ala, Thr 95 Thr/Pro
	020	MDR	4	>32	4	2	4	2	1	2	Ser 531 Leu	none	C -15 T	none	Asp 94 Ala
	049	MDR	32	>32	>128	>128	16	0.25	0.125	0.25	His 526 Arg	none	C -15 T	A 1400 G	none
OM-V06	044	XDR	4	1	>128	64	64	16	8	32	His 526 Ser	none	none	none	Asp 94 Gly
	042	XDR	64	8	>128	16	32	8	4	8	Ser 531 Leu	none	none	none	none

* MIC could not be determined because the isolate perished before we received.

We described characteristics of the cases with OM-V02, OM-V03, and OM-V04 in Table 5. About OM-V02, the age distribution of patients was wide and seven patients were younger than 60 years old. The earliest patients (Nos 019 and 073) were reported in 2002 and the last one (No. 063) was reported in 2008. In 11 patients, seven patients (005, 019, 050, 051, 082, 095, and 004) were newly diagnosed as MDR-TB. As to OM-V03, the age distribution of patients was the age of between 30 and 89, the period when patients reported was from 2000 to 2008, and two of four patients were newly diagnosed. Both patients with OM-V04 were newly diagnosed, one was reported in her 30′s in 2002 and the other was reported in her 60′s in 2003.

## Discussion

To understand the transmission dynamics of MDR/XDR-Mtb in Osaka Prefecture and its neighboring region, an area with the highest TB endemicity in Japan, molecular epidemiological analyses of 89 MDR/XDR-Mtb was performed. Overall, our study demonstrated the following features of Mtb found in Osaka Prefecture and its neighboring regions: 1) a high XDR-Mtb to MDR-Mtb ratio (48.5%); 2) a high ratio (88.8%) of Beijing lineage genotypes among MDR-Mtb; 3) the spread of MDR/XDR-Mtb isolates that share distinct MIRU-VNTR patterns.

Our data revealed a high XDR-Mtb to MDR-Mtb ratio (48.5%) in the studied area. The XDR/MDR ratio in Japan, 31.5% [17] is higher than that of other countries [2], [24]. One of the causes for this high ratio seems to be the presence of the MDR-Mtb SC. Two of the MDR-Mtb SC consisted of only XDR-Mtb isolates and one contained nine XDR-Mtb (Table 4). By excluding XDR-Mtb isolates from the MDR-Mtb SC, the XDR/MDR ratio in this study was determined to be 40.0%, which is still significantly higher than the national ratio. From the available information, we cannot specify what factors result in the increase in XDR/MDR ratio in Osaka. In Osaka Prefecture and Japan, the prevalence of TB patients co-infected with HIV is low, indicating that HIV is not a considerable factor involved in this high XDR/MDR ratio.

**Table 5:** Characteristics of cases and mutations of isolates in the MDR-TB SC.

Cluster	MDR isolate No.	MDR/XDR*	Sex	Age(years)	Year of case reported	History of TB	additional mutation
OM-V02	005	XDR	Female	50–59	2004	New	
	010	XDR	Male	50–59	2006	relapsed	
	019	XDR	Male	40–49	2002	New	*katG,gyrA*
	030	XDR	Female	50–59	2007	relapsed	
	050	MDR	Male	20–29	2003	New	
	051	MDR	Female	50–59	2003	New	
	063	XDR	Male	70–79	2008	Unknown	
	073	XDR	Male	70–79	2002	Unknown	
	082	XDR	Female	20–29	2003	New	
	095	XDR	Male	80–89	2005	New	*rpoB*
	004	XDR	Male	90–99	2004	New	
OM-V03	013	XDR	Male	80–89	2000	Unknown	
	029	XDR	Female	30–39	2006	New	*inhA, gyrA*
	061		Male	Unknown	2008	Unknown	
	064	XDR	Male	60–69	2007	New	
OM-V04	002	MDR	Male	60–69	2003	New	*gyrA*
	020	MDR	Male	30–39	2002	New	

* MDR, multidrug resistant, XDR, extensively-drug resistant.

In this study, the percentage of Beijing lineage genotypes found among MDR-Mtb isolates (79/89, 88.8%) was significantly higher than that of population-based non MDR-Mtb (425/550, 77.3%) which was almost same as obtained nationwide in Japan [18]. The Beijing lineage is of particular concern because it has a high predilection for developing drug resistance [24], which may explain the high percentage of the Beijing lineage in MDR-Mtb seen in this study. Beijing strains can be classified into modern and ancient sub-lineages. Although the modern Beijing sub-lineage was reported to be disseminating worldwide [25], the ratio of modern sub-lineage of the 79 MDR-Mtb isolates belonging to Beijing lineage was 24.1%, which is similar to that reported for Japan [26], [27]. These data suggest that there is no discrepancy of frequency of acquiring MDR/XDR phenotypes between ancient and modern sub-lineages.

Although the ratio of cluster-forming isolates in MDR-Mtb was significantly lower than that in population-based non MDR-Mtb (27.0% and 39.3%, respectively), 6 identical clusters were found in MDR-Mtb isolates. It was been reported that some MDR-Mtb isolates form clusters with identical genotypes [9]–[12], [28]. Although our collection of Mtb isolates analyzed in this study did not fully cover all Mtb isolates before 2007, it is notable that four clusters consisting of 20 (22.4%) MDR-Mtb isolates showed unique MIRU-VNTR patterns that were not detected in non-MDR/XDR-Mtb isolates from Osaka Prefecture and neighboring regions. Based on similarities of genotype, drug resistance, and mutations, we regarded all isolates belonging to OM-V02 and OM-V03, and two isolates from OM-V04 (Nos. 002 and 020; Table 4) as clones. Of these 17 isolates, 11 isolates were isolated from newly diagnosed MDR-TB patients. It was shown that at least these 11 cases were caused by infection not by result of inadequate chemotherapy. Seven of 11 cases with OM-V02 Mtb were newly diagnosed, i.e., OM-V02 Mtb isolates likely transmitted. Since insufficient epidemiological information was available, we could not predict whether the cause of the transmission of these infectious MDR-Mtb were due to social behavior of patients. Chronologically, for each OM-V02 clone, although the additional mutation was observed in No.019, it was isolated earlier than other XDR-Mtb isolates. XDR-Mtb isolates 019 and 073 were isolated before the Nos. 050 and 051 from MDR-Mtb cases. These data suggest that some MDR-V02 clones have remained multidrug resistant and some other derivatives had acquired further drug resistance and had become XDR-Mtb during the spread through multiple infectious routes.

In addition, two additional OM-V02 clones were isolated after the study period. Of these, one was isolated from a new MDR-TB case and the other was from a re-infected patient cured of a drug-sensitive Mtb infection (different from OM-V02) two years prior to reinfection. Although the infectious routes of these two additional cases were not specified, these cases support the evidence of expansion by transmission of OM-V02 Mtb. OM-V02 clones may be warned as a big problem in public health, because XDR-Mtb belonging to OM-V02 was highly resistant Mtb (Table 4) and caused XDR-TB in the wide range of ages (Table5). The systematical and rapid test of 2^nd^ line drugs sensitivity and genotyping are needed to detect OM-V02 clones or such drug resistant strains. Furthermore, the improvement of TB control containing enforced management of patients and thorough contact investigation will be needed to prevent expansion of OM-V02 clones or such strains.

Of 89 MDR-Mtb isolates, the most frequent mutation conferring RFP resistance was S531L in *rpoB* (38/89, 42.7%; data not shown), and the D516V mutation in *rpoB* of OM-V02 Mtb was the third most frequent mutation conferring RFP resistance in 89 MDR-Mtb. The S531L mutation in *rpoB* was reported to not cause reduction of fitness, especially in the Beijing lineage [29], [30], and the D516V mutation did not to impair fitness compared with drug-sensitive clinical isolates [31]. Thus, it is conceivable that the combination with the phylogenetic structures of OM-V02 MDR-Mtb and the mutations conferring resistance to OM-V02 carries a relatively low fitness deficit. Although we could not determine the veritable cause of OM-V02 predominance in this study, it is possible that some Mtb clones like OM-V02 retain or strengthen infectiousness and/or have the ability to progress to an active form of the disease following the acquisition of drug resistance.

In spite of the high ratio of OM-V02 among MDR/XDR Mtb isolates, we did not detect non-MDR/XDR-Mtb strains with genotypes the same as OM-V02. Thus, it is likely that a drug-sensitive ancestral clone of OM-V02 or its derivatives have lower fitness cost than OM-V02 MDR/XDR-Mtb strains. This suggests that the well-organized medical treatment of TB patients with multiple drugs in Osaka, Japan might bring about colonization by selected transmissible highly drug-resistant Mtb clones. Thus, this study highlights that the spread of highly drug-resistant Mtb is not only a problem in developing [24] countries, but also in developed countries with excellent medical care systems, such as Japan.
